# A comprehensive model for gynecologic cancer patients' survivorship care

**DOI:** 10.1016/j.gore.2026.102200

**Published:** 2026-08-28

**Authors:** Victoria E. Rodriguez, Rachel Frankenthal, Jessica Walchonski, Beth Y. Karlan, Dana M. Chase

**Affiliations:** Department of Obstetrics & Gynecology, David Geffen School of Medicine, University of California, Los Angeles, Los Angeles, CA, USA

**Keywords:** Gynecologic Cancer, Survivorship, Comprehensive survivorship care

## Abstract

**Objectives:**

The population of gynecologic cancer survivors is rapidly increasing, yet survivorship care remains fragmented and often focused primarily on surveillance for disease recurrence. This review aims to define the key domains shaping survivorship and propose a comprehensive, patient-centered model for gynecologic oncology.

**Methods:**

Our narrative reviews and synthesizes current evidence and clinical perspectives to examine gynecologic cancer survivorship. We organize the analysis around four clinically relevant pillars: menopause management, sexual health and function, psychosocial health, and nutrition and physical activity. Evidence was summarized and gaps in care are identified to inform a comprehensive survivorship framework.

**Results:**

Gynecologic cancer survivors experience complex and interrelated long-term sequelae. Treatment-induced menopause contributes to substantial symptom burden and increased risk of chronic disease. Sexual dysfunction is highly prevalent yet frequently underrecognized in clinical care. Psychosocial challenges, including depression, anxiety, fear of recurrence, and financial toxicity, are common and persistent. Nutrition and physical activity influence quality of life, physical functioning, and survival outcomes, although gynecologic cancer-specific evidence remains limited. Deficiencies in routine screening and assessment across survivorship domains, clinician knowledge and training, and access to multidisciplinary interventions contribute to unmet survivorship needs.

**Conclusions:**

Survivorship care in gynecologic oncology requires a structured, holistic, and multidisciplinary approach that extends beyond surveillance for disease recurrence. Integrating menopause care, sexual health support, psychosocial services, and lifestyle interventions into routine care is essential to address unmet needs and improve long-term outcomes. A comprehensive model centered on these four pillars provides a practical framework for screening, intervention, and multidisciplinary survivorship care aimed at improving long-term quality of life and health outcomes.

## Highlights

• There is a growing population of gynecologic cancer survivors
• Comprehensive survivorship care is warranted for gynecologic cancer survivors
• There are four key pillars that are central to gynecologic survivorship care

## 1 Introduction

Gynecologic cancers include malignancies of the cervix, ovary and fallopian tube, uterus and endometrium, vagina, and vulva. Together, gynecologic cancers represent the second most common group of cancers affecting women in the United States (Siegel et al., 2025). With rising incidence rates and continued improvements in early detection and treatment, the number of gynecologic cancer survivors continues to increase year over year. As of January 2025, there were approximately 1.5 million gynecologic cancer survivors in the United States, representing nearly 14% of all female cancer survivors (Wagle et al., 2025). This number is expected to increase to 1.7 million by 2035 (Wagle et al., 2025). Accordingly, gynecologic cancer survivors represent a growing population of women with distinct long-term survivorship needs that have often been overlooked and warrant increased study and a comprehensive approach to survivorship care.

While survivorship care has gained increased recognition across oncology, gynecologic cancer survivors face discrete challenges stemming from anatomic, hormonal, and sexual consequences of their disease and treatment. These cancers directly affect reproductive organs and may profoundly impact sexual function, body image, fertility, and hormonal health, intersecting with key aspects of identity, intimacy, and psychosocial well-being (del Carmen and Rice, 2017; Westin et al., 2016; Campbell et al., 2019). Survivors may also experience chronic pain, fatigue, bowel or bladder dysfunction, and limitations in physical functioning, all of which interfere with daily activities and quality of life (Westin et al., 2016; Campbell et al., 2019). Beyond physical sequelae, many survivors report ongoing psychosocial distress, fear of recurrence, and challenges maintaining work, family, and social roles (Campbell et al., 2019). Despite these complex needs, survivorship care for gynecologic cancers remains fragmented, inconsistently delivered, and often overly focused on surveillance for disease recurrence rather than on recovery and long-term well-being.

Gynecologic cancer survivorship is complex, multidimensional, and shaped by the intersection of patient-level factors and structural and social determinants. Comprehensive survivorship care requires a structured, holistic, and patient-centered approach that addresses both acute and long-term sequelae of cancer and its treatment. Four pillars are central to the survivorship care of gynecologic cancer survivors including menopause management, sexual health and function, psychosocial health, and nutrition and physical activity. Here, we examine each of these pillars, summarize current evidence, and outline a comprehensive model for survivorship care in gynecologic oncology.

## 2 Menopause management

### 2.1 Acute symptom burden and long-term health implications

Approximately 30–50% of gynecologic cancer survivors are premenopausal or perimenopausal at diagnosis and many experience abrupt, treatment-induced menopause following surgery, chemotherapy, and/or radiation therapy (del Carmen and Rice, 2017). Compared with natural menopause, treatment-induced menopause is often associated with more severe vasomotor symptoms (VMS), genitourinary syndrome of menopause (GSM), cognitive impairment, and sleep disturbance (del Carmen and Rice, 2017). These symptoms contribute to sexual dysfunction, psychological distress, and diminished quality of life (The North American Menopause Society (NAMS), 2023; Huffman et al., 2016). In addition to symptom burden, premature estrogen deprivation is associated with an increased risk of cardiovascular disease, osteoporosis, stroke, dementia, mental health conditions, sexual dysfunction, and all-cause mortality (Villa et al., 2024). Routine assessment and management of menopausal symptoms and long-term health consequences should therefore be incorporated into survivorship care, with early intervention aimed at improving quality of life and reducing long-term morbidity. Validated tools such as the Menopause Rating Scale (MRS) and the Menopause-Specific Quality of Life (MENQOL) questionnaire may be used to assess symptom burden and the impact of menopause on quality of life (Dolye et al., 2018; Blümel et al., 2018). Suggested screening tools and assessment recommendations are summarized in Table 1.

**Table 1:** Suggested Assessment and Screening Recommendations for Gynecologic Cancer Survivorship Care.

Survivorship Domain	What to Screen For	Suggested Tool(s)	Suggested Referrals
Menopause Management	Vasomotor symptoms, genitourinary syndrome of menopause, sleep disturbance, menopausal symptom burden	Menopause Rating Scale (MRS); Menopause-Specific Quality of Life Scale (MENQOL)	Menopause and/or Hormone Specialists
Sexual Health and Function	Sexual desire, arousal, orgasm, vaginal dryness, dyspareunia, relationship concerns	Brief Sexual Symptom Checklist for Women; Arizona Sexual Experience Scale (ASEX); Female Sexual Function Index (FSFI)	Menopause and/or Hormone Specialists; Pelvic Floor Physical Therapy; Sexual Health; Mental Health
Psychological Distress	Depressive symptoms, anxiety symptoms, overall distress, fear of recurrence	Patient Health Questionnaire-9 (PHQ-9); Generalized Anxiety Disorder-7 (GAD-7); NCCN Distress Thermometer (NCCN DT); Cancer Worry Scale (CWS)	Mental Health
Financial Toxicity	Financial burden, financial distress, healthcare affordability concerns	COmprehensive Score for Financial Toxicity (COST)	Social Work
Nutrition	Weight loss, malnutrition, nutritional risk, healthy eating	Mini Nutritional Assessment (MNA); Malnutrition Screening Tool (MST); Malnutrition Universal Screening Tool (MUST); Nutritional Risk Screening-2002 (NRS-2002); NUTRISCORE, Patient-Generated Subjective Global Assessment Short Form (PG-SGA SF); Healthy Eating Index (HEI)	Registered Dietician
Physical Activity and Function	Physical function, performance status, fatigue	PROMIS Physical Function (PROMIS PF); Eastern Cooperative Oncology Group Performance status (ECOG)	Rehabilitation Specialists; Physical Therapy

Notes: Suggested timing is intended as a practical framework and should be individualized according to cancer type, treatment exposure, symptom burden, recurrence status, and patient preferences. Assessment should occur at diagnosis, throughout treatment, at survivorship visits, at least annually, and additional screening whenever new symptoms emerge or major treatment transitions occur. Positive screening results should prompt further evaluation and referral to appropriate specialists.

### 2.2 Hormone therapy for gynecologic cancer survivors

Hormone therapy (HT) remains the most effective treatment for VMS and GSM and also mitigates some of the long-term health consequences of premature estrogen deprivation. For eligible survivors with bothersome symptoms, HT should be considered a first-line treatment option (Taylor et al., 2024; Sinno et al., 2020; Chambers et al., 2020). Despite growing evidence supporting HT use in many gynecologic cancer populations, fear and misinformation continue to influence practice patterns, with fewer than two-thirds of clinicians reporting prescribing hormones to gynecologic cancer survivors even in situations where evidence supports safety (McDowell et al., 2024).

Current evidence suggests that many gynecologic cancer survivors can safely use HT when treatment decisions are individualized according to cancer type, histology, stage, and recurrence risk. Systemic HT is generally considered acceptable for many survivors of ovarian, endometrial, cervical, vaginal, and vulvar cancers (Taylor et al., 2024; Sinno et al., 2020). Importantly, available studies have not demonstrated increased recurrence risk or adverse survival outcomes among epithelial ovarian and early-stage, low risk endometrial cancers receiving HT (von Kartaschew et al., 2026; Eeles et al., 2015; Barakat et al., 2006; Shim et al., 2014). In contrast, caution is warranted in survivors with tumors known to be hormone responsive (Taylor et al., 2024; Sinno et al., 2020). HT is generally not recommended for low-grade serous ovarian cancer, advanced or recurrent endometroid and granulosa cell tumors, advanced and recurrent endometrial cancers, or uterine sarcomas because of tumor biology and limited safety data (Taylor et al., 2024; Sinno et al., 2020). Cancer-specific recommendations regarding systemic and vaginal hormone therapy are summarized in Table 2.

**Table 2:** Systemic and Vaginal Hormone Therapy Recommendations by Gynecologic Cancer Type.

	Primary Cancer(s)	Acceptable	Caution	Not Recommended
Systemic Hormones	**Ovarian, Fallopian Tube, Primary Peritoneal**	High grade serous; Endometrioid Stage I; Clear Cell; Mucinous; Germ Cell; Borderline (no invasive implants)	Endometroid Stage II+; Granulosa Cell Stage I; Borderline (peritoneal implants, microinvasive disease, residual disease, recurrence)	Low grade serous; Granulosa cell Stage II+
	**Endometrial**	Stage I/II, Grade 1–2 Endometrioid Carcinomas	Stage I/II Grade 3 Endometrioid Carcinomas; Stage I/II Serous; Clear Cell; Undifferentiated	All Stage III/I/V; Recurrent
	**Uterine Sarcoma**			Leiomyosarcoma; Endometrial stromal sarcoma
	**Cervical, Vulvar, and Vaginal**	All		
Vaginal Hormones	**Ovarian, Fallopian Tube, Primary Peritoneal**	All subtypes except low grade serous	Low grade serous Stage II+	
	**Endometrial**	All Stage I/II (except sarcomas)	All Stage III/IV; Recurrent	
	**Uterine Sarcoma**			Leiomyosarcoma; Endometrial stromal sarcoma
	**Cervical, Vulvar, and Vaginal**	All		

References: Chambers 2020; Sinno et al., 2020; Taylor et al., 2024

Notes: Uterine cancer recommendations are based on REFERENCESO 2009 staging in alignment with cited references.

### 2.3 Prescribing considerations

Estrogen therapy is FDA approved for VMS, GSM, osteoporosis prevention, and premature menopause, and is frequently prescribed off-label for symptoms including mood changes, brain fog, sleep disturbance, and arthralgias (Levy and Simon, 2024; Kaufman et al., 2025; Quaile et al., 2025). Transdermal estradiol is generally preferred because it has not been associated with increased risks of venous thromboembolism, stroke, or adverse lipid effects in observational studies (Glynne and Simon, 2026; Cho et al., 2023). Available systemic estrogen, progestogen, combination hormone, vaginal hormone formulations and dosing recommendations are summarized in Table 3.

**Table 3:** FDA-Approved Menopausal Hormone Therapies: Formulations and Dosing.

Therapy Category	Route/Formulation	Dose
Systemic Estrogen Therapy ^a;1^		
Oral	17β-Estradiol	Low: 0.5 mg/day Moderate: 1.0 mg/day High/Premature Menopause: 1.5–2.0 mg/day
	Conjugated estrogens	Low: 0.3–0.45 mg/day Moderate: 0.625 mg/day High/Premature Menopause: 0.9–1.25 mg/day
Transdermal Patch (17β-Estradiol) – Weekly or Twice Weekly	Estradiol patch	Low: 0.025 mg Moderate: 0.0375–0.05 mg High/Premature Menopause: 0.075–0.1 mg
Transdermal Gel (17β-Estradiol) - Daily	Gel, 0.75 mg/pump	Low: 0.75 mg (1 pump) Moderate: 1.5 mg (2 pumps) High/Premature Menopause: 2.25 mg (3 pumps)
	Gel, 0.52 mg/pump	Low: 0.52 mg (1 pump) Moderate: 1.04 mg (2 pumps) High/Premature Menopause: 1.56 mg (3 pumps)
	Gel packet	Low: 0.25–0.5 g Moderate: 0.75–1.0 g High/Premature Menopause: 1.25 g
Transdermal Spray (17β-Estradiol) - Daily	Spray, 1.53 mg/actuation	Low: 1.53 mg (1 spray) Moderate: 3.06 mg (2 sprays) High/Premature Menopause: 4.59 mg (3 sprays)
Vaginal Ring – Systemic (replaced every 90 days)	Estradiol acetate ring	Moderate: 0.05 mg/day High/Premature Menopause: 0.1 mg/day

Progestogen Therapy (Daily Continuous Dosing)^a^		
Oral	Micronized progesterone	Low: 100 mg/day Moderate: 100 mg/day High/Premature Menopause: 200 mg/day
	Medroxyprogesterone acetate	Low: 2.5 mg/day Moderate: 2.5 mg/day High/Premature Menopause: 5 mg/day
	Norethindrone acetate	Low: 2.5 mg/day Moderate: 2.5 mg/day High/Premature Menopause: 5 mg/day
	Norethindrone	Low: 0.35 mg/day Moderate: 0.35 mg/day High/Premature Menopause: 0.7 mg/day
	Drospirenone	Low: 4 mg/day Moderate: 4 mg/day High/Premature Menopause: 4 mg/day
IUD	Levonorgestrel (52 mg)	Low: Any estrogen dose Moderate: Any estrogen dose High/Premature Menopause: Any estrogen dose

Progestogen Therapy (Cycle Dosing: 12–14 days/month)^a^		
Oral	Micronized progesterone	Low: 200 mg/day Moderate: 200 mg/day High/Premature Menopause: 200 mg/day
	Medroxyprogesterone acetate	Low: 5 mg/day Moderate: 5 mg/day High/Premature Menopause: 5 mg/day
	Norethindrone acetate	Low: 2.5 mg/day Moderate: 2.5 mg/day High/Premature Menopause: 5 mg/day
	Norethindrone	Low: 0.35–0.7 mg/day Moderate: 0.35–0.7 mg/day High/Premature Menopause: 0.7 mg/day

Combination Estrogen/Progestogen Products^a;2^		
Oral	17β-Estradiol / micronized progesterone	0.5 mg / 100 mg; 1.0 mg / 100 mg (continuous combined)
	17β-Estradiol / norethindrone acetate	0.5 mg / 0.1 mg; 1.0 mg / 0.5 mg (continuous combined)
	17β-Estradiol / drospirenone	0.5 mg / 0.25 mg; 1.0 mg / 0.5 mg (continuous combined)
	Conjugated estrogens / MPA	0.3/1.5, 0.45/1.5, 0.625/2.5, 0.625/5 mg (continuous combined)
	Conjugated estrogens / MPA (cyclic)	0.625 mg / 5 mg (MPA days 15–28) (sequential cyclic)
	Conjugated estrogens / bazedoxifene	0.45 mg / 20 mg (continuous combined)
Transdermal Patch – Weekly or Twice Weekly	17β-Estradiol / levonorgestrel	0.045 mg / 0.015 mg (continuous combined – weekly)
	17β-Estradiol / norethindrone acetate	0.05/0.14 mg; 0.05/0.25 mg (continuous combined – twice weekly)

Vaginal Hormone Therapy ^b^		
Vaginal Creams	17β-estradiol 0.01% (0.1 mg/g)	Starting Dose: 0.5–1 g daily × 2 weeks Maintenance Dose: 0.5–1 g, 1–3×/week Typical Serum Estradiol (pg/mL): Variable (∼3–5)
	Conjugated estrogen (0.625 mg/g)	Starting Dose: 0.5–1 g daily × 2 weeks Maintenance Dose: 0.5 g, 1–3×/week Typical Serum Estradiol (pg/mL): Variable
Vaginal Inserts	17β-estradiol inserts	Starting Dose: 4 or 10 μg daily × 2 weeks Maintenance Dose: 1 insert twice weekly Typical Serum Estradiol (pg/mL): 3.6 (4 μg), 4.6 (10 μg)
	Estradiol hemihydrate tablets	Starting Dose: 4 or 10 μg daily × 2 weeks Maintenance Dose: 1 insert twice weekly Typical Serum Estradiol (pg/mL): ∼5.5
	Prasterone (DHEA) inserts	Starting Dose: 6.5 mg daily Maintenance Dose: 1 insert daily Typical Serum Estradiol (pg/mL): ∼5
Vaginal Rings	Silicone polymer ring — 2 mg estradiol	Starting Dose: 7.5 μg/day × 3 months Maintenance Dose: 1 ring every 3 months Typical Serum Estradiol (pg/mL): ∼8
Oral Tablet	Ospemifene	Starting Dose: 60 mg daily Maintenance Dose: 60 mg PO daily

References: ^a^ Quaile et al., 2025; ^b^ Kaufman et al., 2025

Notes: ^1^ Doses should be titrated to symptom response. For premature and early menopause, high doses targeting physiologic, premenopausal levels are standard; ^2^ All combination products provide endometrial protection. Bazedoxifene acts as a SERM (estrogen antagonist in the uterus and breast), eliminating the need for a separate progestogen. MPA = medroxyprogesterone acetate.

Survivors with a uterus require a progestogen to prevent endometrial hyperplasia and malignancy, including those who have received pelvic radiation therapy (Barnhill et al., 1985). Although progestogens are not required for endometrial protection in survivors without a uterus, progesterone may provide additional benefits for sleep and mood (Belelli et al., 2022). Whether a progestogen should be added following hysterectomy for endometrial cancer remains uncertain and should be individualized (Hodis and Mack, 2022). Importantly, micronized progesterone has not been associated with an increased risk of breast cancer (Taylor et al., 2024; Skeith and Bates, 2026).

For survivors experiencing premature menopause, guidelines recommend physiologic hormone replacement until at least age 52 years to support long-term health and quality of life (Taylor et al., 2024; von Kartaschew et al., 2026). For all other survivors, the lowest effective dose should be titrated according to symptom relief (Faubion et al., 2022). HT may be continued indefinitely if it continues to align with the survivor's health goals and no new contraindications emerge (Faubion et al., 2022; Sriprasert et al., 2026). Absolute contraindications to HT include unexplained abnormal uterine bleeding, pregnancy, cirrhosis, active hormone-sensitive cancers, active stroke, venous thromboembolism, and myocardial infarction (Faubion et al., 2022).

### 2.4 Non-hormonal interventions

Effective non-hormonal interventions are available for survivors who decline HT or for whom HT is contraindicated (The North American Menopause Society (NAMS), 2023). Management should be individualized according to the survivors' symptom profile and treatment goals. FDA-approved therapies for VMS include fezolinetant, elinzanetant, and paroxetine. Selective serotonin reuptake inhibitors (SSRIs) and serotonin and norepinephrine reuptake inhibitors (SNRIs), including venlaxafine, desvenlaxafine, and escitalopram, as well as gabapentin and oxybutynin, may also be used to reduce VMS (The North American Menopause Society (NAMS), 2023; Denlinger et al., 2017). Non-pharmacologic interventions such as cognitive behavioral therapy, hypnosis, acupuncture, exercise/physical activity, yoga, and lifestyle modifications have also demonstrated efficacy and may be used alone or in combination with pharmacologic therapies (The North American Menopause Society (NAMS), 2023; Denlinger et al., 2017).

Sleep disturbance should be addressed concurrently, particularly when VMS contribute to insomnia. More than 80% of gynecologic cancer survivors report sleep disruption, which negatively affects fatigue, cognition, mood, cardiovascular health, metabolic health, immune function, and mortality (Fox et al., 2022; Fernandez-Mendoza and Vgontzas, 2013). Evidenced-based interventions include cognitive behavioral therapy for insomnia, sleep-restriction therapy, relaxation therapy, and management of night sweats (The North American Menopause Society (NAMS), 2023). Pharmacologic options such as zolpidem, zaleplon, ramelteon, suvorexant and trazodone may also be considered when clinically appropriate. A summary of evidenced-based non-hormonal interventions for menopausal symptoms, sleep disturbance, and GSM is provided in Table 4.

**Table 4:** Non-Hormonal Pharmacologic and Non-Pharmacologic Interventions and Management for Menopausal Symptoms, Sleep Disturbance, and Sexual Health.

Symptom/Concern	Pharmacologic	Non-Pharmacologic
Vasomotor Symptoms	FDA-Approved (Fezolinetant; Elinzanetant; Paroxetine); SSRIs/SNRIs (Venlafaxine; Desvenlafaxine; Escitalopram); Other Agents (Gabapentin; Oxybutynin)	Cognitive Behavioral Therapy; Hypnosis; Acupuncture; Exercise/Physical Activity; Yoga; Lifestyle Modifications
Sleep Disturbance	Zolpidem; Zaleplon; Ramelteon; Suvorexant; Trazodone	Cognitive Behavioral Therapy for Insomnia; Sleep-restriction therapy; Relaxation therapy; Management of night sweats
Vaginal Dryness & Dyspareunia	Vaginal/vulvar moisturizers; Topical 4% aqueous lidocaine (applied before intercourse); Ospemifene	Water- or silicone-based lubricants (with sexual activity)
Radiation-Associated Sexual Dysfunction & Vaginal Stenosis	Vaginal estrogen	Vaginal dilator therapy; Penetrative intercourse; Sexual aids (e.g., vibrators); Pelvic floor therapy; Relaxation training; Manual therapy; Biofeedback
Psychological & Relationship Issues Related to Sexual Health		Mindfulness; Yoga; Meditation; Cognitive Behavioral Therapy; Psychosexual Therapy (Individual or Couple-Based)

References: Archer et al., 2019; Bhinder et al., 2025; Chambers et al., 2020; Delinger et al., 2018; Fernandez-Mendoza & Vgontzas, 2013; Fox et al., 2022; Ghaderi et al., 2019; Huffman et al., 2016; Pennacchini et al., 2024; Shi et al., 2020; Smith et al., 2022; the North American Menopause Society (NAMS), 2023

Notes: Interventions should be individualized according to symptom profile, treatment goals, and contraindications to hormone therapy.

### 2.5 Counseling

All survivors at risk for treatment-induced menopause should receive counseling and proactive management planning prior to treatment (Taylor et al., 2024). Shared decision-making should incorporate symptom burden, cancer histology and stage, recurrence risk, available safety data, long-term health implications of estrogen deprivation, and survivor preferences. Current guidelines recognize that quality of life considerations may lead some patients to pursue HT despite relative contraindications (Taylor et al., 2024). In these situations, treatment decisions should be individualized and guided by informed shared decision-making regarding the potential risks and benefits.

## 3 Sexual health and function

Sexual health concerns are highly prevalent among patients with gynecologic malignancies and should be recognized as a fundamental component of survivorship care. Sexual well-being is a core dimension of health-related quality of life and is strongly linked to emotional, relational, and physical well-being (Mishra et al., 2021). Compared with cancer-free individuals, gynecologic cancer survivors experience substantially higher rates of sexual dysfunction that may persist for months to years after treatment (Mishra et al., 2021). More than half of female survivors of breast, gynecologic, and colorectal cancers report long-term adverse effects on sexual function following treatment, with rates of sexual dysfunction approaching 80% among survivors of cervical cancer (Smith et al., 2022; Qian et al., 2023). Among ovarian cancer survivors, fewer than half remain sexually active after treatment and among those who remain sexually active report lower levels of sexual pleasure and greater discomfort than healthy controls (Liavaag et al., 2008). Sexual dysfunction is multifactorial and may result from surgery, chemotherapy, radiation, hormonal therapy, treatment-induced menopause, and psychological distress. Despite its prevalence and impact, sexual health remains underrecognized and undertreated in routine oncologic care (Bhinder et al., 2025). Given the multifactorial nature of sexual dysfunction in gynecologic cancer survivors, a multidisciplinary approach incorporating gynecologic oncology, menopause care, pelvic floor rehabilitation, mental health support, and sexual health expertise is often required to optimize outcomes.

### 3.1 Screening and assessment

The National Comprehensive Cancer Network recommends routine screening for sexual health concerns in all gynecologic cancer survivors, regardless of sexual activity status (Bhinder et al., 2025). Because sexual dysfunction commonly develops during treatment, screening should begin at diagnosis and continue throughout treatment and surveillance. Many patients report that sexual concerns are not addressed by their healthcare providers, while others are hesitant to initiate these discussions themselves (Girard et al., 2024). These findings underscore the importance of proactively incorporating sexual health assessment into survivorship care, normalizing conversations about sexual function, and offering evidence-based interventions or referral to appropriate specialists when concerns are identified (Girard et al., 2024). Several validated screening tools are available, including the Brief Sexual Symptom Checklist for Women, the Arizona Sexual Experience Scale (ASEX), and the Female Sexual Function Index (FSFI), which can facilitate identification of patient-specific concerns and guide individualized treatment strategies (Smith et al., 2022). Additional screening recommendations for sexual health assessment are summarized in Table 1.

### 3.2 Management of vaginal dryness and dyspareunia

Vaginal dryness and dyspareunia are among the most common sexual health concerns following gynecologic cancer treatment and may lead to avoidance of sexual activity while negatively impacting interpersonal relationships and overall quality of life (Smith et al., 2022). Treatment-induced hormonal disruption and damage to vaginal tissues are primary contributors to these symptoms (Huffman et al., 2016; Mishra et al., 2021). Management begins with either hormonal or non-hormonal therapies and should be evaluated using a personalized approach. In women who have no contraindications, vaginal estrogen can be used to significantly improve symptoms of vaginal dryness and dyspareunia (Huffman et al., 2016). Available vaginal hormone formulations include creams, inserts, tablets, and rings, with dosing information are summarized in Table 3.

Although randomized trials evaluating vaginal estrogen safety in gynecologic cancer survivors are lacking, observational data have not demonstrated an increased risk of recurrence among uterine, cervical and ovarian cancer survivors using vaginal estrogen (Chambers et al., 2020). Vaginal estrogen has minimal systemic absorption thus, experts generally consider it safe for survivors of cervical, vaginal, vulvar, and ovarian cancers, as well as those with early-stage, low-risk uterine cancers (Taylor et al., 2024). As uterine cancers often recur in the vagina, caution is warranted in survivors of advanced, recurrent, or aggressive uterine cancers. Vaginal estrogen is not recommended for uterine sarcoma survivors because of the highly estrogen-sensitive nature of these tumors (Taylor et al., 2024). In these situations, alternative non-hormonal interventions should be used proactively to reduce unnecessary suffering. Regular use of vaginal or vulvar non-hormonal moisturizers at least three times weekly, in combination with water- or silicone-based lubricants during sexual activity, can improve vaginal hydration and reduce dyspareunia (Smith et al., 2022). Topical 4% aqueous lidocaine applied before intercourse has also demonstrated benefit, reducing pain by 88% compared with 33% among placebo-treated patients (Smith et al., 2022). Additional pharmacologic and non-pharmacologic interventions for vaginal dryness and dyspareunia are summarized in Table 4.

For patients whose symptoms remain inadequately controlled with non-hormonal measures, patients who are not candidates for vaginal hormone therapy, or patients who prefer to avoid hormones, ospemifene, an oral selective estrogen receptor modulator with agonist activity in the vulva and vagina, offers an alternative treatment option (Archer et al., 2019). Ospemifene dosing is summarized in Table 3. Clinical trials evaluating ospemifene in post-menopausal women experiencing vaginal dryness demonstrated significant improvement in vaginal epithelial maturation, vaginal pH, and sexual function and is generally well tolerated (Archer et al., 2019). Importantly, women with a history of malignancy within the previous 10 years were excluded from these trials, limiting the generalizability of these data to cancer survivors. However, limited retrospective data in gynecologic cancer survivors have also demonstrated improvement in sexual function without evidence of increased endometrial thickening or cancer recurrence (Pennacchini et al., 2024). Although these preliminary findings are reassuring and may help inform clinical decision-making, larger studies with longer follow-up are needed to establish its safety and efficacy in gynecologic cancer survivors before definitive recommendations can be made.

### 3.3 Radiation-associated sexual dysfunction

Pelvic radiation therapy can have profound and lasting effects on sexual function through both treatment-induced ovarian insufficiency and direct injury to the vaginal tissues. Radiation-associated fibrosis, adhesions, and vaginal stenosis may result in dyspareunia as well as shortening and narrowing of the vaginal canal which can cause difficulty with pelvic examinations and/or penetrative intercourse (Fernandez-Mendoza and Vgontzas, 2013; Mishra et al., 2021). Vaginal stenosis is a common consequence of pelvic radiation, with prospective studies demonstrating grade 2 stenosis in approximately 29% of patients and grade 3 stenosis in 3.6% within six months of treatment (Smith et al., 2022).

Several physical interventions may aid in vaginal rehabilitation. Because these changes may develop early after treatment and become progressively more difficult to reverse, preventive counseling and early vaginal rehabilitation should be incorporated into routine survivorship care. Vaginal dilators are the most studied intervention to reduce the risk of adhesions and stenosis (Bhinder et al., 2025). Numerous cancer organizations recommend initiating vaginal dilation 2–4 weeks following completion of radiation therapy, while continued sexual activity may provide similar benefits when feasible (Bhinder et al., 2025). Adjunctive use of sexual aids, such as vibrators, may further enhance arousal and help prevent tissue adhesions (Mishra et al., 2021; Bhinder et al., 2025). The addition of vaginal estrogen to consistent dilator use can offer synergistic benefits (Pitkin and VanVoorhis, 1971). Dilators work to counteract the fibrotic contracture leading to vaginal narrowing, while estrogen works to promote epithelial proliferation (Pitkin and VanVoorhis, 1971). Counseling regarding these interventions before radiation therapy may improve adherence, facilitate timely initiation of vaginal rehabilitation, and promote earlier recognition and management of treatment-related sexual dysfunction (Denlinger et al., 2017; Pitkin and VanVoorhis, 1971). It is also crucial that these interventions are continued indefinitely as radiation induced changes are typically chronic and progressive (Pitkin and VanVoorhis, 1971). A summary of interventions for radiation-associated sexual dysfunction and vaginal stenosis is provided in Table 4.

### 3.4 Pelvic floor rehabilitation

Pelvic floor dysfunction also contributes substantially to sexual health concerns among gynecologic cancer survivors. Weakness or imbalance of the pelvic floor musculature may contribute to dyspareunia, muscle spasm, decreased sensation, and difficulty achieving orgasm (Huffman et al., 2016). Pelvic floor physical therapy aims to improve muscle strength, coordination, and blood flow while reducing muscle tension. Therapeutic approaches may include targeted exercises, relaxation training, manual therapy, biofeedback, and vaginal dilator therapy (Huffman et al., 2016). Pelvic floor rehabilitation has been shown to improve genito-pelvic pain, sexual dysfunction, and pelvic floor muscle strength and endurance for cancer survivors (Denlinger et al., 2017). Pelvic floor physical therapy should therefore be considered for survivors experiencing dyspareunia, pelvic floor dysfunction, or treatment-related sexual pain.

### 3.5 Psychological and relationship factors

Psychological and emotional factors are important contributors to sexual dysfunction in gynecologic cancer survivors. A cancer diagnosis and its associated treatments may lead to anxiety, depression, altered body image, and fear of recurrence, all of which can negatively impact sexual desire, arousal, and satisfaction (Fernandez-Mendoza and Vgontzas, 2013; Mishra et al., 2021; Liavaag et al., 2008). Evidence-based interventions include cognitive behavioral therapy, psychosexual therapy, and other psychological approaches (Shi et al., 2020; Roussin et al., 2023). Psychological interventions have been associated with improvements in sexual function and depressive symptoms among cervical cancer survivors (Shi et al., 2020). Psychosexual counseling, particularly in a couple-based format, has also been shown to improve sexual function, relationship satisfaction, and psychological outcomes for both patients and partners (Roussin et al., 2023). Additional supportive strategies, including mindfulness, yoga, and meditation may provide adjunctive benefit by reducing stress and anxiety (Denlinger et al., 2017). Psychosexual counseling and cognitive behavioral therapy should be considered when psychological distress, altered body image, relationship challenges, or fear of recurrence contribute to sexual dysfunction. Relevant psychological and relationship-focused interventions are summarized in Table 4.

## 4 Psychosocial health

### 4.1 Psychological distress

Emotional well-being and psychological distress including depression, anxiety, and fear of cancer recurrence represent another core pillar of survivorship care. Depression and anxiety are highly prevalent among gynecologic cancer survivors, with prevalence estimates ranging from 20% to more than 70% depending on cancer type, treatment exposure, and timing of assessment (Westin et al., 2016; Mccorkle et al., 2006; Sanjida et al., 2021; Beesley et al., 2020; Zach et al., 2024; Hodgkinson et al., 2007; Galica et al., 2022). Fear of recurrence is similarly prevalent, affecting approximately one-quarter to one-third of gynecologic cancer survivors and representing one of the most commonly reported supportive care needs (Hodgkinson et al., 2007; Galica et al., 2022; Mell et al., 2022; Osann et al., 2023; Krok-Schoen et al., 2018). Thus, psychological distress is common and may persist long after treatment completion, highlighting the importance of addressing mental health as a routine component of survivorship care.

Biologic, demographic, psychosocial, and clinical factors contribute to adverse mental health outcomes among gynecologic cancer survivors. Treatment-induced hormone deficiency may increase psychological vulnerability, while loss of fertility and disruption of desired childbearing have been associated with greater depression and distress (Canada and Schover, 2012). Younger age, low socioeconomic status, unemployment or work disruption, poor social support, cancer-related symptoms, and comorbid conditions have also been consistently associated with worse emotional health among survivors (Westin et al., 2016; Beesley et al., 2020; Zach et al., 2024; Messelt et al., 2021). Fear of cancer recurrence is more common among younger survivors, those with current disease, those experiencing depression or anxiety, and those closer to diagnosis (Mell et al., 2022; Osann et al., 2023; Krok-Schoen et al., 2018). Conversely, positive coping strategies such as problem-focused coping, emotional processing, and emotional expression have been associated with fewer depressive symptoms (Siwik et al., 2020). Mindfulness-based interventions, peer-support programs, and lifestyle interventions such as home based exercise have been associated with reductions in depression, anxiety, and fear of recurrence, as well as improvements in quality of life (Le et al., 2024; Cohen et al., 2022; Cartmel et al., 2021a; Zhou et al., 2017).

### 4.2 Financial toxicity

Financial toxicity frequently compounds psychological distress and represents a significant survivorship concern. Financial toxicity (i.e., objective financial burden such as high out-of-pocket costs, medical debt, or difficulty paying for care) and related financial stress (i.e., perceptions of financial strain) have been consistently associated with greater distress and poorer quality of life among gynecologic cancer survivors (Benedict et al., 2022; Smith et al., 2024). Across studies, 17% to 44% of survivors report moderate to severe financial toxicity, while up to 69% report financial stress (Benedict et al., 2022; Smith et al., 2024). Younger age, single marital status, lower socioeconomic status, racial and ethnic minority status, and public or self-pay insurance have been associated with worse financial outcomes (Benedict et al., 2022; Smith et al., 2024; Aviki et al., 2022). Survivors frequently report unmet needs related to out of pocket expenses (Galica et al., 2022). Interventions specifically targeting financial toxicity and financial stress among gynecologic cancer survivors remain limited, despite its documented impact on distress and quality of life. Early identification of financial concerns and referral to social work services, financial counseling programs, patient assistance programs, and community resources may help mitigate the impact of financial toxicity on survivorship outcomes.

### 4.3 Screening and assessment

Routine psychosocial screening should be incorporated into survivorship care beginning at diagnosis and continuing throughout treatment and survivorship. Additional assessment may be warranted during major treatment transitions, recurrence, or when new symptoms emerge. However, many gynecologic cancer survivors report unmet psychosocial care needs (Galica et al., 2022), thus proactive screening, normalization of mental health discussions, and timely referral for supportive services are therefore critical components of survivorship care.

Several validated screening tools are available to facilitate identification of psychosocial concerns. Commonly used instruments include the Patient Health Questionnaire-9 (PHQ-9) for depressive symptoms (Fulton et al., 2024), the Generalized Anxiety Disorder-7 (GAD-7) for anxiety (Fulton et al., 2024), the NCCN Distress Thermometer (NCCN DT) to assess overall distress (Ownby, 2019), the Cancer Worry Scale (CWS) to assess fear of cancer recurrence (Custers et al., n.d.), and the COmprehensive Score for Financial Toxicity (COST) measure to assess financial toxicity in cancer populations (Pangestu and Rencz, 2023). These tools can assist in identifying survivors who may benefit from further evaluation, intervention, or referral. Survivors with clinically significant symptoms identified through screening should be referred for mental health evaluation and evidenced-based psychological interventions when appropriate. Early identification of depression, anxiety, fear of cancer recurrence, and financial toxicity, coupled with timely referral to appropriate supportive services may improve emotional well-being, quality of life, and overall survivorship outcomes. A summary of recommended psychosocial and financial toxicity screening tools and referral pathways is provided in Table 1.

## 5 Nutrition and physical activity

Among gynecologic cancer survivors, treatment-related side effects frequently compromise nutritional status and physical functioning. These effects can result in muscle wasting, malnutrition, weight changes, frailty, cancer-related fatigue, and decreased health-related quality of life (Westin et al., 2016; Campbell et al., 2019; Morton et al., 2023). Furthermore, sarcopenia, defined as the loss of skeletal muscle mass and function, may develop during treatment and persist into survivorship, impacting long-term outcomes including survival (Morton et al., 2023). Despite the clinical importance of these issues, nutrition and physical activity remain among the most understudied domains of gynecologic cancer survivorship, and many existing recommendations are extrapolated from broader cancer survivorship populations (Kenkhuis et al., 2024).

### 5.1 Screening and assessment

Nutrition and physical function assessment should begin at diagnosis and continue throughout treatment and survivorship (*CA Cancer J. Clin.*, 2022). Routine screening for nutritional risk, sarcopenia, changes in body composition, physical inactivity, and functional decline should be incorporated into survivorship care visits. Survivors identified being at risk should be referred to trained nutrition professionals, rehabilitation specialists, physical therapists, or structured exercise programs when appropriate (*CA Cancer J. Clin.*, 2022). Several validated nutrition screening tools are available, including the Mini Nutritional Assessment (MNA), Malnutrition Screening Tool (MST), Malnutrition Universal Screening Tool (MUST), Nutritional Risk Screening-2002 (NRS-2002), NUTRISCORE, the Patient-Generated Subjective Global Assessment Short Form (PG-SGA SF), and the Healthy Eating Index (HEI) (Trujillo et al., 2024; Kaur et al., 2025). Validated tools such as PROMIS Physical Function (PROMIS PF) and the Eastern Cooperative Oncology Group Performance status (ECOG), can be used to assess physical function and performance status (Jensen et al., 2015; Azam et al., 2019). Suggested nutrition and physical activity screening tools and referral pathways are summarized in Table 1.

### 5.2 Nutrition recommendations

Given the limited gynecologic-cancer specific evidence base, current dietary recommendations are largely informed by the American Cancer Society (ACS) guidelines for cancer survivors (*CA Cancer J. Clin.*, 2022; Rock et al., 2022). These guidelines emphasize routine nutritional assessment, maintenance of a healthy body weight, preservation of lean muscle mass, and adherence to a healthy dietary pattern (Rock et al., 2022). Survivors should be encouraged to consume diets rich in vegetables, fruits, whole grains, and legumes while limiting processed meats, red meats, refined grains, and highly processed foods (*CA Cancer J. Clin.*, 2022; Rock et al., 2022).

Maintaining adequate protein intake is particularly important given the high prevalence of functional decline among cancer survivors. At minimum, survivors should meet recommended dietary allowances for vitamins and minerals and consume sufficient protein (>1 g of protein per kilogram of body weight per day) to support preservation of muscle mass and physical function (Rock et al., 2022). When nutritional requirements cannot be met through diet alone, oral nutritional supplementation should be considered (Rock et al., 2022). The European Society for Parenteral and Enteral Nutrition recommends protein intake of approximately 1.0 to 1.5 g/kg/day and caloric intake of 25–30 kcal/kg/day, with individualization based on age, comorbidities, treatment effects, and nutritional status (Muscaritoli et al., 2021).

Among women with endometrial cancer, obesity at diagnosis has been associated with an increased all-cause mortality (Rock et al., 2022), highlighting the importance of weight management strategies that prioritize metabolic health and preservation of muscle mass. Observational studies among ovarian cancer survivors further suggest that dietary quality may influence long-term outcomes. Higher Empirical Dietary Inflammatory Pattern (EDIP) scores, reflecting more pro-inflammatory diet patterns, have been associated with increased risk of all-cause and ovarian cancer-specific mortality, whereas lower EDIP scores, associated with greater consumption of anti-inflammatory foods, have been associated with improved survival outcomes (Sasamoto et al., 2022). Declines in overall diet quality from pre- to post-diagnosis were similarly associated with worse survival outcomes (Sasamoto et al., 2022). Although limited, these findings support recommendations favoring healthy dietary patterns as part of comprehensive survivorship care.

### 5.3 Physical activity recommendations

Physical activity is an essential component of survivorship care and should be addressed routinely alongside nutritional health. The ACS guidelines found that higher levels of physical activity are associated with lower all-cause mortality among gynecologic cancer survivors, while acknowledging the limited number of intervention studies within this population (Rock et al., 2022). Current recommendations include 150 to 300 min per week of moderate-intensity activity or 75–150 min of vigorous-intensity activity, or a combination of the two, in addition to at least two sessions of muscle-strengthening activities (*CA Cancer J. Clin.*, 2022). Strength training deserves particular emphasis in gynecologic cancer survivorship because treatment-related sarcopenia and loss of physical function are common and may persist long after treatment completion (Morton et al., 2023). Preservation and restoration of lean muscle mass should therefore be considered central goals of survivorship care alongside weight management (Morton et al., 2023; *CA Cancer J. Clin.*, 2022).

Although intervention studies remain limited, available evidence suggests that exercise programs are feasible and acceptable among gynecologic cancer survivors, but their impact on long-term behavioral and clinical outcomes remains uncertain. Baseline data from the ongoing Lifestyle Intervention for Ovarian Cancer Enhanced Survival (LIVES) trial demonstrated that approximately half of survivors failed to meet national physical activity recommendations, highlighting a substantial gap between survivorship recommendations and actual health behaviors (Thomson et al., 2023). Similarly, the PADVOA trial, which incorporated both aerobic and resistance exercise, demonstrated improvements in fat-free mass, physical functioning, and fatigue over time; however, no significant differences were observed between the intervention and usual care groups (Kenkhuis et al., 2024) Likewise, a Fitbit-based physical activity intervention among endometrial cancer survivors demonstrated feasibility and modest short-term improvements in activity levels, however these benefits were not sustained long term and did not result in significant improvements in most health or quality of life outcomes (Rahimy et al., 2021). Additional studies evaluating exercise interventions among ovarian cancer survivors were associated with significant reductions in depressive symptoms and improved physical health related quality of life (Zhou et al., 2017; Cartmel et al., 2021b). Lastly, a home-based strength-training intervention demonstrated feasibility and acceptability among endometrial cancer survivors and preliminary findings showed clinical improvements in several physical function assessments; however authors encouraged further testing for long-term outcomes (Gorzelitz et al., 2022; Gorzelitz et al., 2023). While these studies support the incorporation of routine physical activity for gynecologic cancer survivors, there is a need for further research and implementation strategies that promote sustained long-term engagement in exercise.

## 6 Recommendations for comprehensive survivorship care

What emerges from this evidence is a clear mandate for structured, comprehensive, and multidisciplinary survivorship care that moves beyond exclusive surveillance for recurrent and new cancers. Routine screening for menopausal symptoms, sexual health concerns, psychological distress, financial toxicity, and nutritional and physical functioning should be embedded in survivorship programs from the time of diagnosis. Clinician education, particularly regarding hormone therapy safety, sexual health communication, and the social determinants of survivorship, is essential and currently inadequate across specialties. Table 5 provides actionable recommendations for the implementation of comprehensive survivorship care in gynecologic oncology. Moving forward, comprehensive survivorship care must treat menopause and sexual health as core determinants of long-term health and well-being, while systematically addressing psychological distress, financial toxicity, and health behaviors such as nutrition and physical activity. Such an integrated, multidisciplinary approach is essential to improving quality of life and optimizing outcomes for gynecologic cancer survivors across the survivorship continuum.

**Table 5:** Recommendations for Comprehensive Gynecologic Cancer Survivorship Care.

Key Pillars	Recommendations
Menopause Management	**Normalize menopause management as foundational survivorship care.** • Menopause should be recognized as a core determinant of quality of life, health span, and longevity in gynecologic cancer survivors. • Survivors experiencing induced menopause from cancer treatment should receive comprehensive counseling prior to treatment. • Clinicians should routinely assess menopausal symptoms and long-term health risks and offer hormone therapy when safe, alongside evidence-based nonhormonal options. • All providers caring for survivors should receive menopause specific education. • Survivorship programs should ensure access to comprehensive menopausal care through integrated services or trusted referrals.
Sexual Health and Function	**Center sexual health as a standard component of survivorship care.** • Sexual health must be proactively addressed through clear provider patient communication, routine standardized screening, and early referral to multidisciplinary services. • Survivorship care should assess the full spectrum of contributors to sexual dysfunction, including physical, hormonal, and psychosocial factors, and offer evidenced-based interventions. • Sexual health should be discussed early and revisited throughout survivorship, rather than addressed only when distress becomes severe.
Psychosocial Health	**Systematically screen for mental health concerns and financial toxicity.** • Given the high prevalence and clinical impact of depression, anxiety, fear of recurrence, and financial toxicity, survivorship programs should embed routine screening and response pathways for these concerns. • Care models should integrate mental health support, peer programs, and financial navigation services, with tailored interventions for high-risk groups defined by age, socioeconomic status, and treatment history. • In addition to individual level support and interventions, structural approaches such as financial navigation, cost transparency and system level advocacy are necessary to reduce financial toxicity.
**Nutrition and Physical Activity**	**Integrate nutrition and physical activity into survivorship from diagnosis onward.** • Survivorship care must actively support nutrition health and physical functioning by incorporating routine nutritional assessment, individualized dietary counseling, and physical activity guidance beginning at diagnosis. • Access to dieticians and rehabilitation specialists should be standard, with particular attention to interventions that improve adherence and long-term behavior change. • Greater investment in gynecologic cancer specific trials is needed to define optimal dietary and physical activity strategies.

## 7 Conclusion

The need for comprehensive survivorship care is clear; however, the evidence base underpinning many of these recommendations remains uneven. As advances in treatment and early detection have led to a growing population of gynecologic cancer survivors, unmet survivorship needs have become more visible and consequential. Addressing these unmet needs also require meaningful investment in gynecologic cancer-specific survivorship research, including adequately powdered clinical trials designed to optimize both survival and long-term outcomes. Future efforts must also prioritize populations most often underserved, including younger survivors, racial and ethnic minorities, and individuals with lower socioeconomic status. Ultimately, the goal of survivorship care is not merely survival, but the restoration and preservation of quality of life, health span, and well-being. By integrating these four pillars into a cohesive, patient-centered model of care, gynecologic oncology can ensure that survivors not only survive but thrive.

## Funding disclosure

Victoria E. Rodriguez was supported by the 10.13039/100000002National Institutes of Health, 10.13039/100000054National Cancer Institute T32 Patient-Centered Outcomes Research Training in Urologic and Gynecologic Cancers Program (T32CA251072).

## CRediT authorship contribution statement

**Victoria E. Rodriguez:** Writing – review & editing, Writing – original draft. **Rachel Frankenthal:** Writing – review & editing, Writing – original draft. **Jessica Walchonski:** Writing – review & editing, Writing – original draft. **Beth Y. Karlan:** Writing – review & editing, Supervision. **Dana M. Chase:** Writing – review & editing, Writing – original draft, Supervision.

## Declaration of competing interest

The authors declare that they have no known competing financial interests or personal relationships that could have appeared to influence the work reported in this paper.
